# Case Report: Recurrent anti-glutamic acid decarboxylase 65 antibody-associated encephalitis in a child

**DOI:** 10.3389/fimmu.2026.1729994

**Published:** 2026-01-21

**Authors:** Pingping Tian, Juan Li, Yong Liu, Xiufang Ding, Hongwei Zhang

**Affiliations:** 1 Neurology department, Children’s Hospital Affiliated to Shandong University, Jinan, Shandong, China; 2 Neurology department, Jinan Children’s Hospital, Jinan, Shandong, China

**Keywords:** anti-GAD65 antibody, encephalitis, pediatrics, recurrence, rituximab

## Abstract

**Objective:**

To explore the clinical characteristics of recurrent pediatric anti-glutamic acid decarboxylase 65 (GAD65) antibody-associated autoimmune encephalitis and to enhance understanding of this disease.

**Methods:**

We conducted a retrospective analysis of the clinical data of a pediatric patient with recurrent anti-GAD65 antibody-associated encephalitis.

**Results:**

The patient was a 7-year-old boy who presented with slurred speech and epileptic seizures in June 2024. Brain magnetic resonance imaging(MRI)demonstrated multiple abnormal signal intensities in the bilateral frontal, temporal, parietal, and occipital cortical regions. Electroencephalogram (EEG) revealed focal epileptiform discharges and status epilepticus. His clinical condition improved following treatment with methylprednisolone and intravenous immunoglobulin. In November 2024, the child presented with a decline in mental status, generalized weakness, increased somnolence, reduced mobility and social interaction, gait instability, as well as accompanying headache and vomiting. Brain MRI demonstrated inflammatory involvement of the right cerebellar hemisphere. The cerebrospinal fluid (CSF) analysis revealed a white blood cell count of 17×10^6^/L. Anti- GAD antibody IgG was positive in both serum and cerebrospinal fluid, with a titer of 1:100.The diagnosis of anti-GAD65 antibody-associated encephalitis was well-established. The clinical symptoms of the child showed improvement following the administration of methylprednisolone and immunoglobulin therapy. Following discharge, monthly administration of immunoglobulin therapy was initiated. In February 2025, the child presented with tremors in the right foot, a repeat MRI scan of the cranial revealed an increase in multiple abnormal signals within the brain compared to prior imaging studies, indicating the presence of new lesions. Concurrently, testing for anti-GAD65 antibodies was positive, with titers of 1:1 in CSF and 1:32 in serum. These findings were interpreted as indicative of a relapse of anti-GAD65 antibody-associated encephalitis. Following the administration of a repeat methylprednisolone pulse therapy, the child’s symptoms showed improvement, and sequential treatment with rituximab was subsequently initiated. During the follow-up visit in September 2025, the pediatric patient remained asymptomatic with no evidence of disease recurrence.

**Conclusions:**

We present a detailed case report of recurrent anti-GAD65 antibody-associated encephalitis in a pediatric patient, characterized by complex and variable clinical manifestations. Rituximab demonstrated therapeutic effectiveness in managing this recurrent condition.

## 1 Introduction

Autoimmune encephalitis (AE) is an inflammatory disorder of the central nervous system mediated by anti-neuronal antibodies (1).Since the initial identification of anti-N-methyl-D-aspartate receptor (NMDAR) encephalitis in 2007 (2),numerous autoantibodies targeting neuronal cell surface or synaptic proteins have been identified. In addition to anti-NMDAR antibodies, anti-leucine-rich glioma-inactivated 1 (LGI1) antibodies and anti-GAD65 antibodies are among the most frequently observed autoantibodies (1).Specifically, anti-GAD65 antibody-associated encephalitis accounts for approximately 13.2% of all autoimmune encephalitis cases (3) and is considered relatively rare in clinical settings. GAD65 is the rate-limiting enzyme responsible for catalyzing the conversion of glutamate, an excitatory neurotransmitter, into γ-aminobutyric acid (GABA), a key inhibitory neurotransmitter in the central nervous system. It functions as an intracellular protease and is highly expressed in GABAergic neurons as well as pancreatic β cells (4–6).Anti-GAD65 antibodies selectively bind to GAD65 expressed in GABAergic neurons, thereby interfering with GABAergic transmission and release, reducing GABA levels, and resulting in neuronal hyperexcitability. This pathological process may give rise to a range of neurological manifestations, including epileptic seizures, altered mental status, behavioral disturbances, cognitive impairment, and ataxia (4, 6, 7). Currently,anti-GAD65 antibody-associated encephalitis is predominantly documented in adult patients, whereas pediatric cases are less frequently reported. This observation is thought to be attributable to both the relatively low incidence of the disease in children and the limited clinical awareness of anti-GAD65 antibody-related encephalitis among clinicians. Currently, the recurrence rate of this type of encephalitis in adult patients is as high as 9.64% (8), whereas documented cases of recurrence in pediatric populations remain rare. A case of recurrent anti-GAD65 antibody-associated encephalitis in a pediatric patient is presented. By reviewing the relevant literature, this report aims to increase awareness among pediatric clinicians regarding this rare condition and to provide insights that may support early diagnosis and timely intervention.

## 2 Case presentation

**Figure 1:**
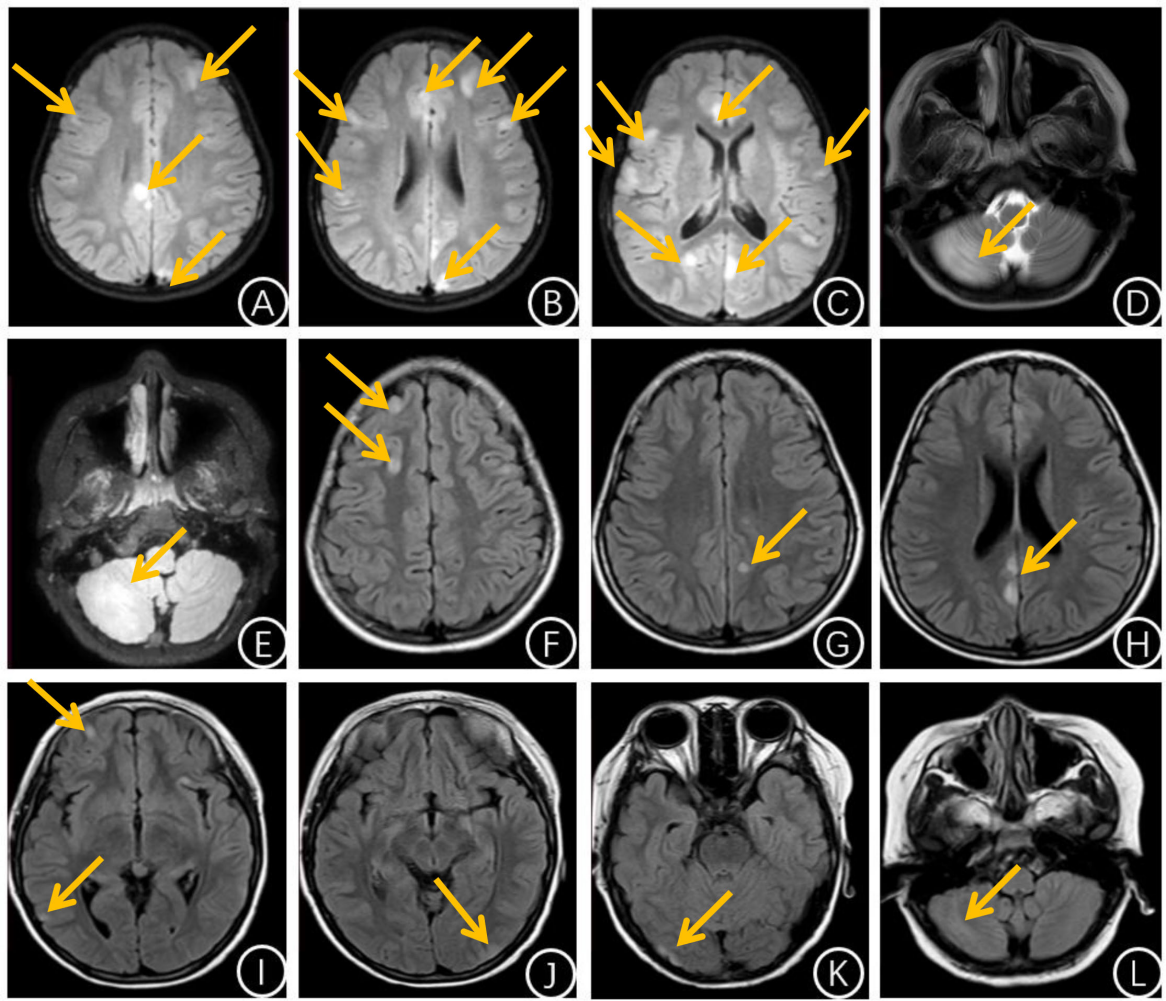
**(A-C)** Brain MRI-FLAIR during the initial acute phase of the child’s illness revealed multiple patchy areas of hyperintensity in the bilateral frontal, parietal, temporal, and occipital regions. Most of these lesions were located in the cortical areas and showed no significant enhancement post-contrast. **(D, E)** Brain MRI during the second acute episode revealed a large patchy area of hyperintensity in the right cerebellar hemisphere on both T2-weighted and FLAIR sequences. **(F-L)** Brain MRI-FLAIR during the third acute episode revealed hyperintensities in the bilateral frontal, temporal, occipital, and parietal lobes, the cingulate gyri, and the right cerebellar hemisphere. The bilateral hippocampi appeared to be involved.

The patient, a 7-year-old male, was admitted to the Department of Neurology at Jinan Children’s Hospital in June 2024 due to a 5-day history of dysarthria and a half-day history of convulsions. Five days prior to admission, the child developed dysarthria without any apparent precipitating factors. Half a day before admission, the patient experienced a seizure episode, characterized by oral commissure twitching, eyelid fluttering, lip smacking, and unresponsiveness to verbal stimuli. The episode lasted approximately 10 minutes and resolved spontaneously. Nine hours later, the child again became unresponsive to external stimuli, accompanied by upward gaze deviation. Additional clinical features were not specifically documented. The second episode lasted approximately half an hour before spontaneous resolution. Following the onset of symptoms, the child received initial treatment at a local hospital. Brain MRI revealed multiple areas of abnormal signal intensity in the bilateral frontal, temporal, parietal, and occipital cortices (As shown in **Figure 1**). During the course of treatment at our institution, the patient exhibited intermittent twitching at the oral commissures. Past Medical History: The patient had a suspected history of head trauma twenty days prior to admission. No significant personal or family medical history was identified. Physical Examination upon Admission: Oral commissure twitching was observed. The patient was alert and in a stable general condition. No abnormal neurological findings were identified. Auxiliary examinations: Cerebrospinal Fluid Analysis: White blood cell count was 5×10^6^/L, protein level was 100 mg/L, and glucose and chloride levels were within normal ranges. Complete blood count, urinalysis, stool examination, liver and kidney function tests, cardiac function tests, and electrolyte levels were all within normal limits. Blood and cerebrospinal fluid tests for infection showed no abnormalities. Serum immunoglobulin M:3.52g/L(Reference range:0.44–1.414g/L), Immunoglobulin G and Immunoglobulin A levels were within normal limits. Serum total T lymphocyte relative count: 49.94% (Reference range: 60.05–74.08%).EEG: Findings indicated an abnormal pediatric EEG pattern with seizure activity. Electroclinical correlation revealed multiple episodes occurring during sleep, with concurrent EEG recordings showing bilateral occipital sharp-slow wave complexes, followed by bilateral occipital low-amplitude spike rhythms and bilateral occipital spike rhythms with increased amplitude, each lasting 1–2 minutes. Additionally, two electroclinical seizure episodes were observed during both wakefulness and sleep. These included focal seizures with preserved consciousness, perioral clonic movements, and one episode fulfilling criteria for status epilepticus. All serum antibodies associated with central nervous system demyelinating diseases were negative. Similarly, antibodies related to autoimmune encephalitis in both serum and CSF were not detected. These central nervous system demyelinating antibodies include: anti-aquaporin 4 (AQP4) antibodies, anti-myelin oligodendrocyte glycoprotein (MOG) antibodies, anti-glial fibrillary acidic protein (GFAP) antibodies, and anti-myelin basic protein (MBP) antibodies. Autoimmune encephalitis-associated antibodies include: anti-glutamate receptor (NMDA type) IgG antibody, anti-glutamate receptor (AMPA1 type) IgG antibody, anti-glutamate receptor (AMPA2 type) IgG antibody, anti-leucine-rich glioma-inactivated protein 1 (LGI1) IgG antibody, anti-gamma-aminobutyric acid B receptor (GABAB) IgG antibody, and anti-contact-associated protein 2 (CASPR2) IgG antibody. Oligoclonal bands in both serum and cerebrospinal fluid were also negative. Based on the child’s clinical manifestations, EEG, and brain MRI, a diagnosis of antibody-negative autoimmune encephalitis is considered. For therapeutic intervention, the patient received intravenous immunoglobulin at a total dose of 2 g/kg, followed by methylprednisolone administered intravenously at 500 mg/day for 3 consecutive days, subsequently reduced to 250 mg/day for an additional 3 days. This was followed by a sequential transition to oral prednisone at a daily dose of 30 mg. The patient presented with status epilepticus and was managed with a sequential treatment regimen consisting of midazolam infusion, intravenous levetiracetam, and oral perampanel to achieve seizure control. On the 6th day of hospitalization, the patient’s oral commissure twitching gradually subsided; however, tremors in the left fingers developed. Concurrently, an EEG was performed, revealing that the background activity was slightly slower than age-appropriate norms. During the waking state, several episodes were recorded, characterized by tremors in the left ring and little fingers, without clear epileptiform patterns. Each episode lasted from several seconds to several minutes, and the exact nature of the events remained undetermined, although subcortical myoclonus was highly suspected. The patient continued oral administration of perampanel and levetiracetam for episode control. The patient was hospitalized for 15 days and discharged following clinical improvement. One month post-discharge, a follow-up brain magnetic resonance imaging (MRI) revealed no abnormal signals in the cerebral parenchymal signal intensity. After discharge, the child continued oral prednisone tablets at 30 mg daily, with a 5 mg reduction every 2 weeks for a total of 3 months of oral therapy. The medication was discontinued in September 2024.

In November 2024, the child presented with a decline in general condition, including lethargy, reduced appetite, reluctance to ambulate and engage in social interaction, and gait instability, accompanied by headache and vomiting. Following admission, repeat brain MRI revealed a large patchy lesion in the right cerebellar hemisphere exhibiting slightly prolonged T1 and prolonged T2 signal intensities, with high signal intensity observed on FLAIR imaging, consistent with an inflammatory lesion (As shown in **Figure 1**). Cerebrospinal Fluid Analysis: White blood cell count was 17×10^6^/L, protein concentration was 119 mg/L, and both glucose and chloride levels were within normal ranges. The EEG demonstrated focal slow-wave activity. Anti-glutamic acid decarboxylase antibody IgG was positive in both serum and cerebrospinal fluid, with a titer of 1:100 (CBA:cell-based assay) in both samples. All other autoantibodies associated with autoimmune encephalitis were negative. Oligoclonal bands were detected in the CSF. Complete blood count, urinalysis, stool examination, liver and kidney function tests, cardiac function tests, and electrolyte levels were all within normal limits. Blood and cerebrospinal fluid tests for infection showed no abnormalities. Free triiodothyronine: 3.17 pmol/L (Reference range: 4.29-6.79 pmol/L), Thyroid-stimulating hormone:0.31 uIU/mL (Reference range: 0.70-4.17 uIU/ml), Free thyroxine within normal range. Thyroglobulin antibody (TGAb) level was 124.64 IU/ml(Reference range: ≤4.1IU/ml); anti-thyroid peroxidase antibody (TPO-Ab) level was 158.85 IU/ml(reference range:0-30IU/ml). Lymphocyte relative counts: Blymphocyte relative count:26.39% (reference range: 10.21-20.12%); total T lymphocyte relative count and natural killer (NK) cell relative count were both within normal limits. Based on the diagnostic criteria for autoimmune encephalitis in 2022, the diagnosis of anti-GAD65 antibody-associated autoimmune encephalitis was established. During hospitalization, chest CT and abdominal contrast-enhanced CT were performed to exclude the presence of neoplastic lesions; however, no significant abnormalities were identified. For therapeutic intervention, intravenous immunoglobulin (total dose 2 g/kg) and methylprednisolone (500 mg/day) were re-administered. The dosage of methylprednisolone was reduced by half every three days for two consecutive reductions, followed by a sequential transition to oral prednisone at a daily dose of 40 mg. The patient was hospitalized for 17 days and discharged following clinical improvement. Following discharge, the patient continued with sequential oral prednisone therapy. Concurrently, the patient returned to the hospital monthly for intravenous immunoglobulin therapy at a dose of 35 grams (1 gram per kilogram).One month after discharge, follow-up cranial MRI demonstrated a significant improvement in the previously observed abnormal signal within the right cerebellar hemisphere.

In February 2025, the child was readmitted to the hospital due to intermittent twitching of the right foot. During the same period, repeat EEG monitoring revealed one episode of electrographic discharge during sleep and several episodes of limb twitching, which were identified as non-epileptic events. Cerebrospinal fluid analysis: white blood cell count was 5×10^6^/L, protein and glucose and chloride levels were within normal ranges. Follow-up cranial magnetic resonance MRI revealed multiple abnormal FLAIR hyperintensities in the bilateral frontal, temporal, parietal, and occipital lobes, as well as the cingulate gyrus and the right cerebellar hemisphere, with bilateral hippocampal involvement (as shown in **Figure 1**). The lesion count was increased compared to prior imaging, indicating the presence of new lesions. The titer of anti-GAD65 antibody in cerebrospinal fluid was 1:1, and the titer in serum was 1:32. All other autoantibodies associated with autoimmune encephalitis were negative. Complete blood count, urinalysis, stool examination, liver and kidney function tests, cardiac function tests, and electrolyte levels were all within normal limits. Blood and cerebrospinal fluid tests for infection showed no abnormalities. Serum antinuclear antibodies were all negative; Serum immunoglobulin M: 3.65 g/L (Reference range: 0.47–2.2 g/L), immunoglobulin G and immunoglobulin A within normal limits. Complement component C3 and C4 within normal limits. Relative B-lymphocyte count: 29.69% (reference range: 10.21–20.12%), relative natural killer (NK) cell count within normal limits. According to the 2022 definition of relapsing autoimmune encephalitis (1), the child developed new symptoms of limb tremors following a period of symptom remission, with an interval exceeding two months. Concurrently, new lesions were identified on cranial MRI. These findings are indicative of a relapse of anti-GAD65 antibody-associated encephalitis. The pediatric patient received intravenous methylprednisolone at a daily dose of 500 mg. After three days, the dosage was tapered to 250 mg per day and maintained for three days. Subsequently, oral prednisone was initiated at a maintenance dose of 30 mg daily. Given the high risk of recurrence in the pediatric patient, rituximab was administered at a daily dose of 400 mg (375mg/m^2^)for two consecutive days following communication with the patient’s family. The same dosage was repeated after a two-week interval. In September 2025 (six months since the last rituximab treatment),follow-up evaluation of the pediatric patient revealed no obvious clinical symptoms and disease recurrence. The clinical manifestations, treatment methods and follow-up results of the three disease courses of the child patient are shown in **Table 1**. The cerebrospinal fluid test results for the three disease stages of the child are shown in **Table 2**.

**Table 1:** Clinical presentation, intervention approaches, and follow-up outcomes of the pediatric patient.

Course of the disease	Main symptoms	Anti-GAD antibody titer (serum/csf)	EEG	Brain MRI	clinical intervention	therapeutic effect
First course of the illness	Slurred speech, status epilepticus	unknown	Abnormal EEG during childhood, electroclinical seizure: Several seizures occurred during sleep. Concurrent EEG showed epileptiform discharges in the bilateral occipital regions. Two seizures occurred during both wakefulness and sleep. Manifestations included focal onset, retained awareness, and perioral clonic seizures. Status epilepticus was observed.	Multiple high-signal areas on FLAIR in the bilateral frontal, temporal, parietal and occipital lobes of the cortex	High-dose methylprednisolone pulse therapy combined with immunoglobulin	Symptoms were relieved, but recurred after a 5-month interval.
First recurrence	Poor mental state, conscious fatigue, increased sleep, and unsteady gait	1:100 (Serum and Cerebrospinal fluid)	Focal slow-wave activity	Large patchy FLAIR hyperintense foci were observed in the right cerebellar hemisphere.	High-dose methylprednisolone pulse therapy combined with immunoglobulin, Monthly immunoglobulin therapy after discharge.	Symptoms were relieved, but recurred after a 2-month interval.
Second recurrence	Limb shaking	Serum: 1:32; Cerebrospinal fluid: 1:1	Interictal discharges were present, characterized by abundant discharges in the left occipital and posterior temporal regions and occasional discharges in the right occipital and posterior temporal regions.	Multiple abnormal FLAIR hyperintense foci were present in the bilateral frontal, temporal, parietal, and occipital lobes, the cingulate gyri, the right cerebellar hemisphere, and the bilateral hippocampi.	High-dose methylprednisolone pulse therapy combined with rituximab treatment	The symptoms were relieved. After a 6-month follow-up, the child had no recurrence.

**Table 2:** Results of routine cerebrospinal fluid tests during the child’s three disease episodes.

Course of the disease	Color of cerebrospinal fluid	Microbial testing	White blood cell count	Single nucleated cell count	Segmented nucleated cell count	Protein quantification(Reference value:150-450mg/L)	Chlorine(Reference value:111-123mmol/L)	Glucose(Reference value:2.8-4.4mmol/L)
First course of the illness	Colorless and transparent	Negative	5×10^6^/L	5个	0个	100	129	3.88
First recurrence	Colorless and transparent	Negative	17×10^6^/L	16个	1个	119	122	3.92
Second recurrence	Colorless and transparent	Negative	5×10^6^/L	5个	0个	165	124	4.25

## 3 Discussion

In 1988, Solimena et al. (9) first identified anti-GAD65 antibodies in a study published in *The New England Journal of Medicine*, which was the first intracellular synaptic antigen antibody to be identified. Anti-GAD65 antibody-associated encephalitis is an autoimmune disorder mediated by anti-GAD65 antibodies. Unlike anti-neuronal cell surface antigen antibody-associated encephalitis, which primarily induces relatively reversible neuronal dysfunction through humoral immune mechanisms (1), previous studies have largely attributed the pathogenesis of anti-GAD65 antibody-associated encephalitis to irreversible neuronal damage or apoptosis mediated by cytotoxic T cells (5, 10, 11). On one hand, GAD-specific CD8+T lymphocytes can destroy GAD-expressing neurons; on the other hand, GAD-specific CD4+T lymphocytes can assist in activating killer cells and B lymphocytes. However, some studies suggested that GAD65 antibodies may have pathogenic effects (12–14). It has been proposed that the GAD65 antigen, which is embedded in synaptic vesicle membranes, may transiently become exposed on the neuronal cell surface during GABA exocytosis by GABAergic neurons, or that antibodies may be internalized via endocytosis and gain access to intracellular antigens (15). These mechanisms could enable pathogenic antibodies to interact with GAD65, thereby disrupting its association with GABA-containing synaptic vesicles, reducing GABA release, and ultimately contributing to disease onset. Anti-GAD65 antibodies can also act on the nerve terminals of GABAergic interneurons to inhibit GABA release, leading to neuronal hyperexcitability (4, 6). Additionally, Thaler FS et al. (16) detected circulating GAD-reactive B cells capable of differentiating into antibody-producing cells in the peripheral blood and bone marrow of patients with neurosyndromes associated with anti-GAD65 antibodies. Therefore, if anti-GAD65 antibodies are directly pathogenic or whether they are just an epiphenomenon for autoimmune disorders that are mediated by CD4+ T cells is still a matter of debate (4).

Anti-GAD65 antibodies in pediatric patients are predominantly associated with limbic encephalitis (LE), extralimbic encephalitis, epilepsy and panencephalitis involving both limbic and extralimbic regions. Among these clinical phenotypes, limbic encephalitis is most common, while panencephalitis affecting both limbic and extra-limbic areas is relatively rare (12, 17–20). The pediatric patients may concurrently present with autoimmune diseases such as diabetes mellitus and autoimmune thyroid disorders (10, 19, 20). Some individuals may exhibit variations in clinical phenotypes may be linked to the specificity of GAD65 antigenic epitopes (3–5). Extralimbic encephalitis exhibits variable clinical manifestations depending on the specific brain regions involved. The presentation may or may not include epileptic seizures, autonomic dysfunction, or signs of brainstem and cerebellar involvement. Cranial MRI may demonstrate hyperintense signals on T2-weighted and FLAIR sequences in the extratemporal lobe cortex and subcortical regions, typically in the absence of contrast enhancement (17, 18). Multiple studies have demonstrated that anti-GAD65 antibody-associated encephalitis frequently manifests with epileptic seizures as the initial clinical presentation (17–19), predominantly of the focal type (17, 21), which may be associated with suppressed gamma-aminobutyric acid (GABA) function in hippocampal neurons and enhanced excitability in the limbic cortex (5, 21). Some pediatric patients may present with status epilepticus (13, 21) or epilepsia partialis continua (EPC) (10). Lin JJ et al (21).performed anti-GAD65 antibody testing in a cohort of 17 pediatric patients with encephalitis complicated by status epilepticus and reported a positivity rate of 35.3%. Furthermore, research has indicated that the production of anti-GAD65 antibodies in patients with anti-GAD65 antibody-associated encephalitis may exhibit a certain latency period, which can extend up to 8 years (18).Therefore, in clinical practice, for pediatric patients presenting with unexplained seizures or status epilepticus, attention should be paid to testing for anti-GAD65 antibodies, including repeated testing when necessary, to facilitate early diagnosis and enable timely immunotherapy, thereby improving patient outcomes. The initial presentation of this patient was primarily characterized by speech disturbances and status epilepticus. Cranial MRI revealed multiple FLAIR hyperintensities in the frontal, temporal, parietal, and occipital cortical regions, suggesting the possibility of anti-GAD65 antibody encephalitis, classified as extralimbic encephalitis. However, anti-GAD65 antibody testing was not performed in this case, which is a deficiency that should be addressed in the clinical management of the patient.

Anti-GAD65 antibody-associated cerebellar ataxia is the second most common anti-GAD65 antibody-associated neurological syndrome. Typical symptoms include gait ataxia, dysarthria, and nystagmus (7). Cranial MRI reveals normal findings or cerebellar atrophy. Prognosis is poorer compared to other clinical phenotypes, but early immunomodulatory intervention can improve outcomes (7, 22). Approximately 80% of patients have coexisting organ-specific autoimmune disorders, such as type 1 diabetes mellitus (T1DM), autoimmune thyroiditis, or pernicious anemia (5). Approximately 25% of patients may exhibit overlapping symptoms with classic SPS (22, 23), and 4.6% of anti-GAD65 antibody-associated encephalitis patients may present with cerebellar ataxia (8), a condition recognized as the overlapping syndrome (24). Cerebellar ataxia is also a common phenotype of paraneoplastic syndrome, necessitating screening for tumors. Commonly associated tumors include small cell or non-small cell lung cancer, neuroendocrine tumors, and others (6). Cerebellar ataxia is relatively rare in pediatric patients with anti-GAD65 antibody-associated neurological syndromes, and to date, no detailed reports exist of overlapping syndromes involving encephalitis. This pediatric patient experienced a recurrence of encephalitis two months after the initial onset and discontinuation of prednisone treatment. Serum and cerebrospinal fluid tests were positive for anti-GAD65 antibodies. Clinical manifestations included unsteady gait without dysarthria or nystagmus. However, the child concurrently exhibited altered mental status, suggesting an overlap syndrome of encephalitis combined with cerebellar ataxia. The child’s cranial MRI revealed cerebellar involvement only, with subsequent development of cerebellar atrophy—a finding not previously reported in pediatric cases of anti-GAD65 antibody-associated encephalitis. This suggests that even in cases where cranial magnetic resonance imaging reveals isolated cerebellar involvement, but clinical manifestations include both cerebellar ataxia and encephalitic symptoms, clinicians should remain vigilant for a possible anti-GAD65 antibody-related overlapping syndrome, and test for anti-GAD65 antibodies. Encephalitis involving both the limbic and extralimbic systems is rare and typically presents with epileptic seizures and disturbances in consciousness. Cranial MRI demonstrates diffuse involvement of the cortical and deep gray matter across both cerebral hemispheres (17). The child’s second recurrence of encephalitis demonstrated whole-brain inflammation involving the hippocampus and cingulate gyrus on cranial MRI, yet clinically presented with involuntary limb movements. The clinical manifestations were milder and less typical than previously reported cases. However, this child experienced recurrent encephalitis episodes within a short timeframe, exhibiting a relapse-remission-relapse-remission pattern, with distinct clinical phenotypes across all three disease courses, indicating the clinical heterogeneity and highly active and aggressive nature of anti-GAD65 antibody-associated encephalitis. In clinical practice, it is essential to differentiate this condition from other forms of autoimmune encephalitis (such as anti-NMDAR encephalitis),viral encephalitis, as well as from multiple sclerosis (MS) and acute disseminated encephalomyelitis (ADEM) in pediatric patients. Each recurrence in this child has presented with milder symptoms than the initial episode, consistent with the typical course of autoimmune encephalitis (AE) relapses (1).

High titers of anti-GAD65 antibodies in serum and CSF and/or evidence of intrathecal synthesis in CSF(presence of oligoclonal bands or an elevated IgG index), are of diagnostic significance (1, 5, 17). During the first recurrence of encephalitis in this pediatric patient, the white blood cell count in the cerebrospinal fluid was only mildly elevated, and IgG oligoclonal bands were detected exclusively in the cerebrospinal fluid. This indicates the production of anti-GAD65 antibodies within the cerebrospinal fluid, supporting the presence of GAD65-specific B cells within the central nervous system rather than impaired blood-brain barrier (BBB) integrity. Hou JY et al. (18) demonstrated that children with encephalitis exhibiting high titers of anti-GAD65 antibodies are more likely to present with initial symptoms of language impairment and ataxia. However, there is currently no consensus on whether antibody titers correlate with disease severity and prognosis. Studies have demonstrated that serum anti-GAD65 antibody titers >500 nmol/L is an independent risk factor for adverse outcomes (7). However, other studies have reported that anti-GAD65 antibody titers may not correlate with disease severity or prognosis across individuals (14, 15, 17, 18). The titer of GAD antibodies may decline following immunotherapy,; however, this reduction is not associated with the clinical phenotype, disease severity, and patient prognosis (6). However, persistently high titers of anti-GAD65 antibodies are typically associated with poor clinical response (17). During the second recurrence of encephalitis in this child, the antibody titers in the serum and CSF (1:32 and 1:1) were lower than those observed during the first relapse (1:100). Nevertheless, clinical symptoms and imaging findings indicated ongoing disease activity. This observation suggests that the absolute antibody titer value does not necessarily exhibit a linear correlation with clinical disease activity. The pediatric patient in this case did not undergo further antibody testing during the initial recovery phase following recurrence of encephalitis, making it impossible to determine the sustained changes in antibody titers. The dynamic change trend of titer, such as a continuous decline, may provide more valuable insights than the absolute value of a single measurement.

Treatment regimens and efficacy vary across different clinical subtypes and syndromes of anti-GAD65 antibody-associated encephalitis. Current treatment approaches primarily derive from case reports or clinical experience, lacking randomized controlled trials (4, 10, 17, 19). The immunotherapy principles for anti-GAD65 antibody-associated encephalitis are largely consistent with those for other types of autoimmune encephalitis. First-line treatments include high-dose corticosteroid pulse therapy, intravenous immunoglobulin, and plasma exchange. However, there is no unified standard for the specific treatment duration. The steroid tapering process requires comprehensive assessment based on the child’s clinical severity, recovery status, and risk of relapse. Current guidelines recommend a total steroid treatment duration of at least six months (1). In this case, the child’s modified Rankin Scale(mRS) scores remained ≤2 during all three disease episodes. The condition improved following first-line immunotherapy. The initial course of corticosteroid treatment for this child lasted approximately three months. The recurrence may be associated with the relatively short duration of corticosteroid therapy. There are currently case reports on second-line immunotherapy for anti-GAD65 antibody-associated encephalitis both domestically and internationally, but large-scale data remain lacking (15, 17, 20, 24–26).In a German study (25) involving 358 cases of autoimmune encephalitis, rituximab treatment was found to be significantly effective in patients with neuronal surface antibody-associated encephalitis, such as anti-NMDAR encephalitis and LGI1 encephalitis, and was associated with a reduced recurrence rate. However, no significant therapeutic effect was observed in patients with anti-GAD65 antibody-associated encephalitis. Nevertheless, it cannot be excluded that delayed initiation of treatment following symptom onset may have influenced these outcomes. Triplett J et al. (26) reported a case of a child with anti-GAD65 antibody-associated encephalitis who presented with status epilepticus and acute encephalopathy. The pediatric patient showed a poor response to first-line immunotherapy, but clinical symptoms improved significantly following the addition of rituximab and cyclophosphamide. Qiu Z et al. (24) found that 15.6% of patients with anti-GAD65 antibody-associated encephalitis who showed no significant improvement after first-line therapy and MMF treatment experienced clinical improvement after switching to RTX therapy. Among them, one patient with limbic encephalitis achieved complete remission. Following the first recurrence of encephalitis in this pediatric patient, maintenance therapy with intravenous immunoglobulin (1 g/kg) was initiated on a monthly basis. However, two months after the initiation of this treatment, the child experienced a recurrence of encephalitis. Following treatment with rituximab after the recurrence, no further relapses occurred, supporting the efficacy of rituximab in preventing recurrence. On the other hand, it suggests that repeated use of immunoglobulin may be less effective than second-line immunomodulatory agents in preventing recurrence. However, the follow-up period for this patient was relatively short, and regular follow-ups are still needed. There is currently no consensus on the duration of steroid therapy and long-term immunosuppressive treatment during remission for patients with relapsed AE. Relevant literature remains limited. Whether steroid therapy for at least 6 months and long-term immunosuppressive therapy for 3 years are applicable to all relapsed patients remains undecided. Treatment should be individualized based on the severity of the patient’s condition at initial presentation, the number of relapses, and adverse drug reactions (27). Anti-GAD65 antibody-associated encephalitis responds less favorably to immunotherapy compared to encephalitis caused by antibodies against neuronal surface proteins such as anti-NMDAR (1, 3, 21, 23, 25). However, 70% of children with anti-GAD65 antibody-associated encephalitis still have a favorable prognosis (8, 15, 18). Research indicates that children with extratemporal lobe encephalitis tend to have a more favorable prognosis, whereas those with temporal lobe encephalitis are more susceptible to developing drug-resistant epilepsy and are at higher risk for hippocampal atrophy and disease recurrence (17). Currently, there is a limited number of research reports on cases of anti-GAD65 antibody-related encephalitis in children, both domestically and internationally, and large-scale studies are still lacking. The factors that influence its prognosis and recurrence remain to be fully elucidated.

In summary, anti-GAD65 antibody-related encephalitis is a rare subtype of AE, characterized by highly heterogeneous clinical manifestations. It may manifest as extralimbic encephalitis, panencephalitis involving both the limbic and extralimbic systems, and Overlap Syndrome of Cerebellar Ataxia with Encephalitis. While the majority of patients respond favorably to immunotherapy, there remains a risk of disease recurrence. Currently, there is limited literature on the recurrence of GAD65 antibody-related encephalitis in pediatric patients both domestically and internationally, and there remains a lack of established experience in its management. In the present case, the child’s recurrence was effectively managed with rituximab; however, the follow-up period was relatively short, and long-term monitoring is warranted. Currently, the understanding of recurrent anti-GAD65 antibody-related encephalitis remains limited. Further investigation is needed to clarify its clinical characteristics, therapeutic approaches, and prognosis.

## Patient perspective

Our family’s journey has been long and arduous. The initial uncertainty of the diagnosis was overwhelming, and watching our child suffer through relapses was heartbreaking. We are deeply grateful to the medical team for their perseverance and for offering rituximab as a potential pathway. While the road to recovery requires patience, we are encouraged by the stability he has maintained since this treatment. Sharing our story is our way of contributing to the understanding of this rare disease and offering solidarity to other families in a similar situation.

## Data Availability

The datasets presented in this article are not readily available because of ethical and privacy restrictions. Requests to access the datasets should be directed to the corresponding author/s.

## Glossary

GAD65: Glutamic acid decarboxylase 65
EEG: Electroencephalogram
MRI: Magnetic resonance imaging
CSF: Cerebrospinal fluid
AE: Autoimmune encephalitis
NMDAR: N-methyl-D-aspartate receptor
LGI1: Leucine-rich glioma-inactivated 1
GABA: γ-Aminobutyric acid
TGAb: Thyroglobulin antibody
TPO-Ab: Anti-thyroid peroxidase antibody
LE: limbic encephalitis
EPC: epilepsia partialis continua
ADEM: Acute disseminated encephalomyelitis
mRS: Modified Rankin Scale
